# Supplementary material for: Quantitative optical nanoscopy of mitochondrial-derived vesicles in neurons classifies pre-peroxisomal and clearing organelles
Source: Nat Commun. 2026 Jan 8;17:419. doi: 10.1038/s41467-025-68160-y (PMC12796351; doi:10.1038/s41467-025-68160-y)
Supplement: Supplementary file 1 — Supplementary Information new [file 41467_2025_68160_MOESM1_ESM.pdf]

Supplementary Figures

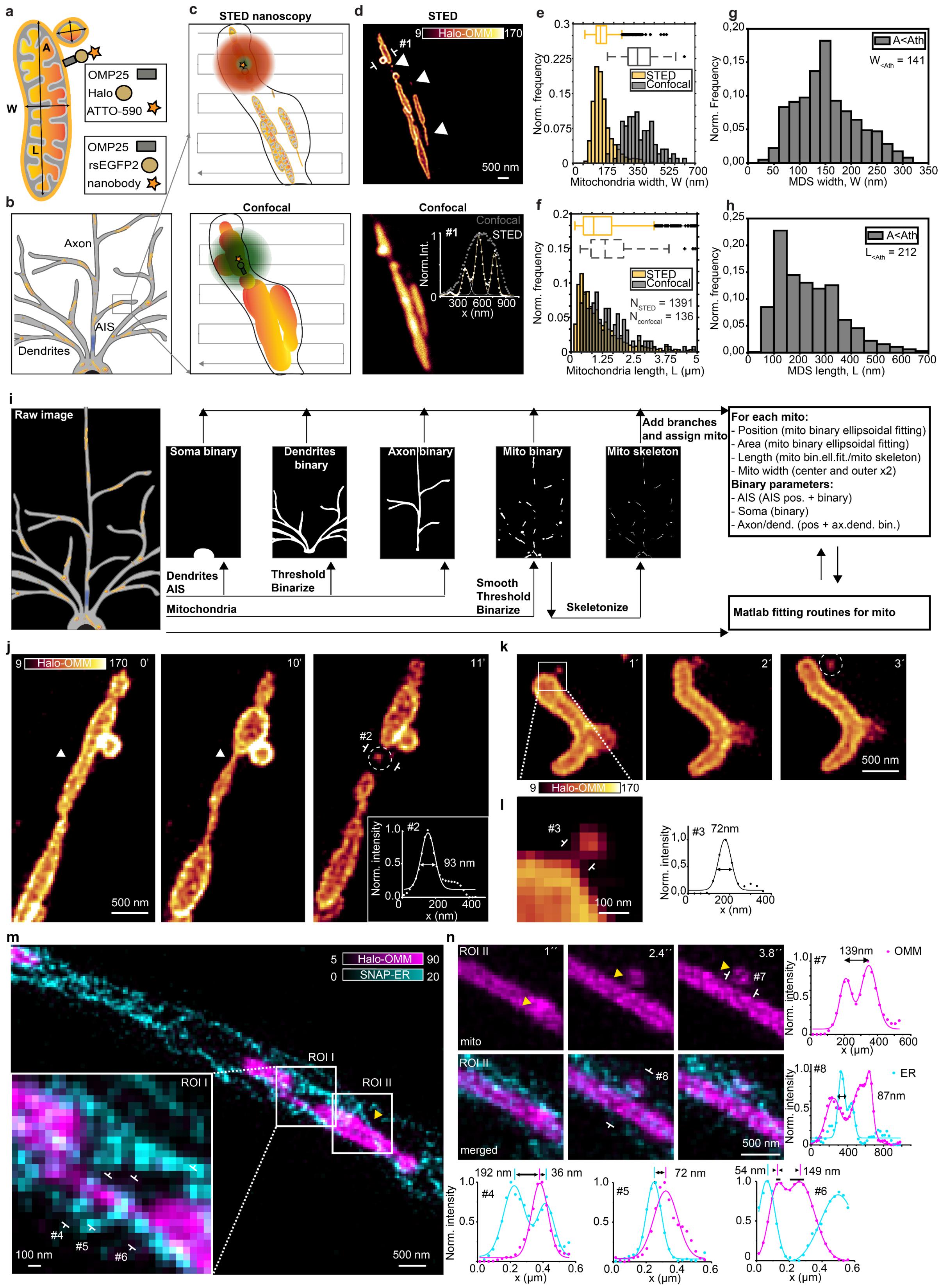

Supplementary Figure 1. Automated analysis of MDSs.

(a) Schematic of an MDS near a mitochondrion showing labelling strategies and the measured parameters: mitochondria width (W), mitochondria length (L) and mitochondria area (A).

(b) Schematic of a neuron with mitochondria in yellow and the axon initial segment (AIS) in cyan.

(c) Schematic of STED and confocal microscopy to identify MDSs expressing fluorescently tagged OMM.

(d) STED versus confocal images of mitochondria and MDSs, showing the outer membrane resolved in STED but unresolved in confocal. Inset: line profile across #1 for confocal and STED.

(e) Histogram of mitochondria width (W, bin = 21nm) with box plot (25th and 75th percentiles, outliers as +) form STED (yellow, N = 1391 organelles) and confocal (grey, N = 136 organelles) images, from 31 neurons, more than 3 independent experiments.

(f) Histogram of mitochondria length (L, bin = 120nm) with box plot (25th and 75th percentiles, outliers as +).

(g) MDS width distribution (histogram, bin= 20 nm), from 21 neurons, 4 independent experiments. $N_{MDVs} = 637$, median = 141 nm.

(h) MDS length distribution (histogram, bin= 50 nm), from 21 neurons, 4 independent experiments. $N_{MDVs} = 637$, median = 212 nm.

(i) Mitography workflow: automated ImageJ analysis generates binary masks of soma, dendrites, axons and mitochondria from three-channel raw or deconvolved images. Mitochondrial parameters, including centroid position, angle (ellipsoidal fitting), area, length, AIS/soma/dendrites assignment, and skeleton-based branch length, are extracted through mask operations. Position and angle are used to obtain line profiles for middle/outer mitochondrial width via MATLAB fitting.

(j) Representative frames of a live-STED time-lapse showing a mitochondrion (Halo-OMM) undergoing a fission and forming of a ~ 90 nm vesicle. The line profile across position #2 is shown in the inset. See Supplementary Movie 1.

(k) Representative frames of a live-STED time-lapse showing the lateral membrane protrusion and MDS formation. See Supplementary Movie 2.

(l) Zoom-in of the vesicle neck and relative line profile, across the denoted line (FWHM = 72 nm).

(m) Representative frame of a two-colour live-STED time-lapse showing two mitochondria (Halo-OMM, red hot), likely undergoing fission, connected by a nanotunnel within an ER network (SNAP-Sec-61β, cyan). The inset zooms in on the nanotunnel, with line profiles (#4-6) highlighting ER-mitochondrial contacts. See Supplementary Movie 3.

(n) Zoom-in to a ROI I in (M) showing vesicle formation over four seconds: membrane accumulation, lateral protrusion, and vesicle pinching. Line profiles across #7 and #8 reveal a thin ER filament (78 nm) at the budding site and the size of the newly formed vesicle.

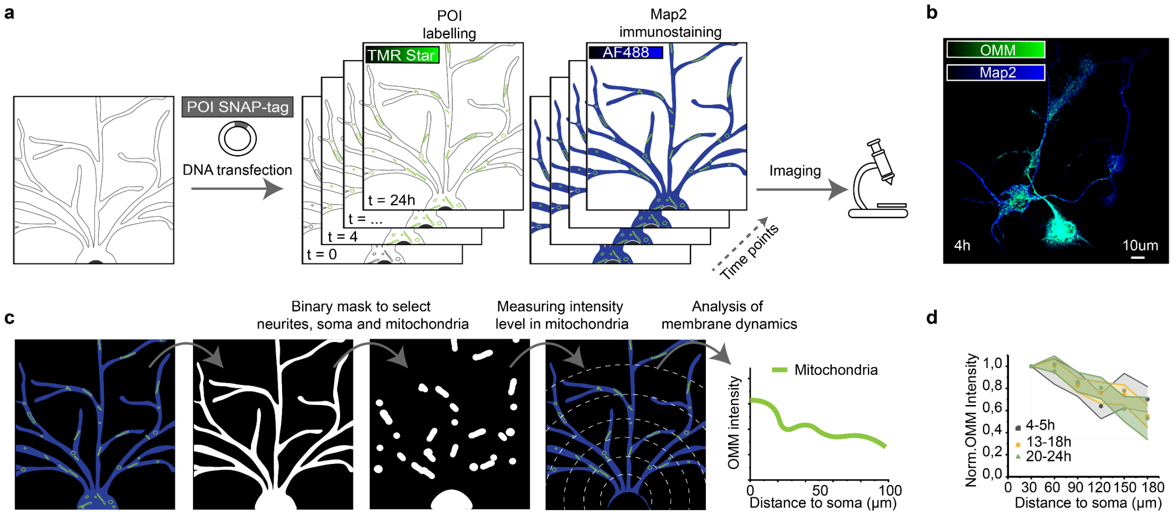

Supplementary Figure 2. Newly synthesized proteins are equally integrated within the neuronal cell.

(a) Workflow scheme of the SNAP-tag protein expression system to follow the exogenous protein distribution over time and space within the full neuron. The SNAP-tag labelling of the protein of interest (POI) with SNAP-TMR-Star-BG ligand is performed at different timepoints after transfection (0, 4, 13, 16, 18, 20 and 24h), followed by fixation and immunostaining of the protein Map2 (blue).

(b) Representative example of a neuronal cell transfected with OMM-SNAP plasmid for 4hours, labelled with SNAP-TMR-Star-BG (OMM, green) and Map2 (blue).

(c) Workflow scheme of the image analysis for the quantification of the exogenous protein distribution over time and space.

(d) Quantification of the SNAP-TMR-Star-BG intensity (OMM), normalized by its intensity in the most proximal mitochondria, measured at different distances from the cell soma, for three different transfection times: 4-5h (grey); 13-18h (yellow) and 20-24h (green). Shaded areas denote 83.4% confidence intervals and thus their overlap corresponds to $p > 0.05$ for t-tests for different means. Data were collected from 34 cells from 7 independent experiments.

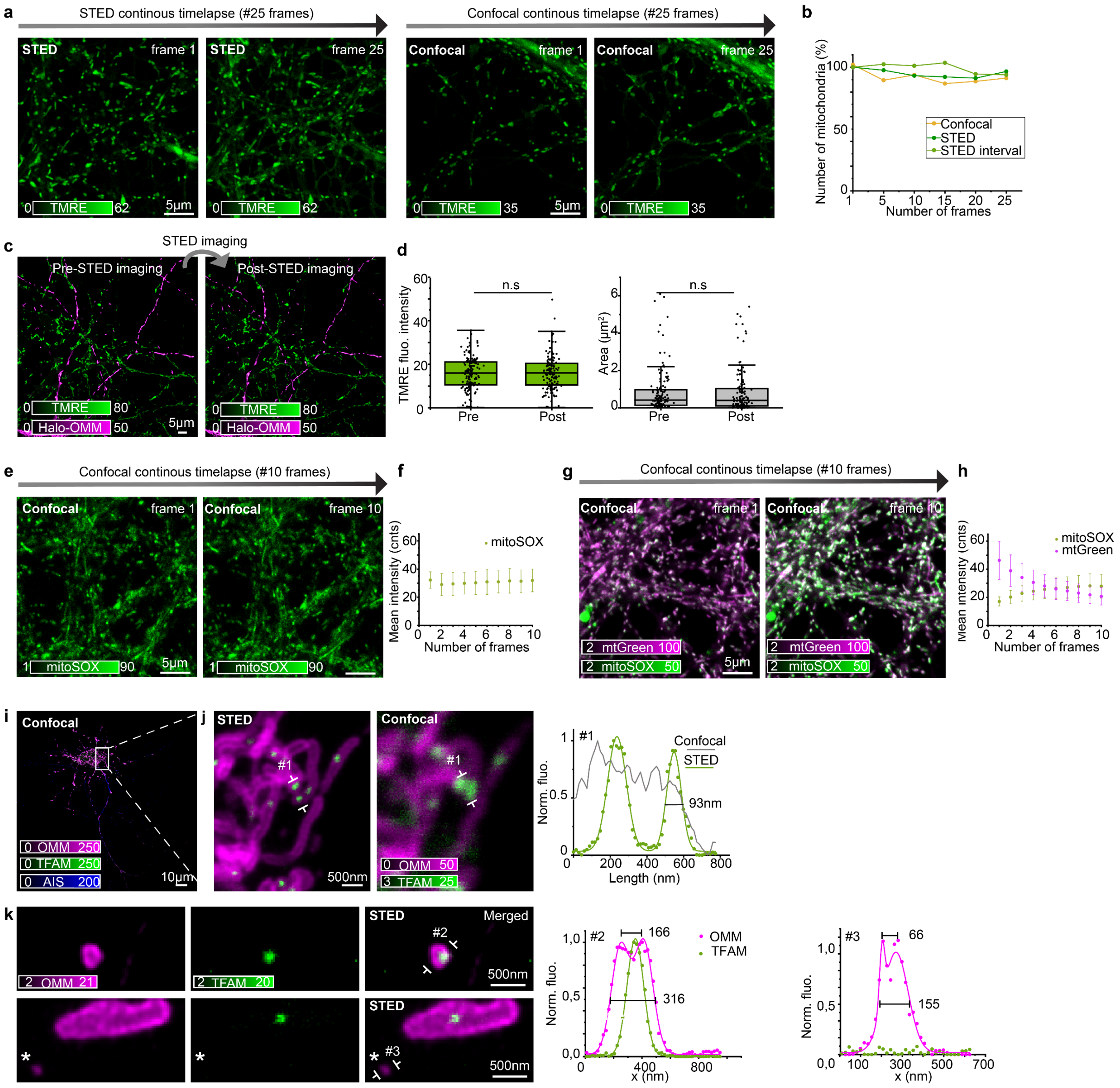

Supplementary Figure 3. STED imaging does not affect mitochondrial membrane potential nor ROS production.

(a) Confocal (right) and STED (left) time-lapse of 25 consecutive frames of neurons where mitochondria were labelled with the membrane potential sensitive dye TMRE. Frames #1 and #25 are shown.

(b) Number of mitochondria identified in each frame, shown as percentage, and normalized to the number of mitochondria identified in frame 1.

(c) Confocal images of mitochondria (Halo-OMM, magenta) and TMRE (green) before and after a conventional STED image acquisition experiment (multiple recordings).

(d) Box plot showing the intensity of TMRE and the mitochondria area, measured before and after the STED image acquisition experiment. Each dot is one mitochondrion, N = 137 (pre), N = 143 (post), from one experiment.

KS test $p_{intensity} = 0.75$; $p_{area} = 0.73$. For the box plot the center line represents the median, the box spans the interquartile range (IQR; 25th to 75th percentiles) and whiskers extend to 1.5× IQR.

(e) Confocal recording of mitochondria labelled with mitoSOX.

(f) Mean intensities measured over 10 consecutive frames. Frames #1 and #10 are shown. Each dot is one frame, mean +/- SD.

(g) Confocal recording of mitochondria labelled with mitoSOX (green) and MitoTracker Green (mtGreen, magenta)

(h) Mean intensities measured for 10 consecutive frames in both channels. Frames #1 and #10 are shown. Each dot is one frame, mean +/-SD.

(i) Confocal image of a neuronal cell where mitochondria (Halo-OMM, magenta), nucleoids (TFAM immunostaining, green) and the axon initial segment (AIS, Pan-neurofascin immunostaining, blue) are labelled.

(j) Two-colour confocal and STED comparison of multiple mitochondria. STED image, in contrast to confocal, allows to discriminate between single nucleoids. The inset shows the line profile of two nucleoids measured across #1.

(k) Two-colour STED images and line profiles, measured across #2 and #3, of two vesicles of different sizes (166 nm and 66 nm), where only the larger one has a nucleoid inside.

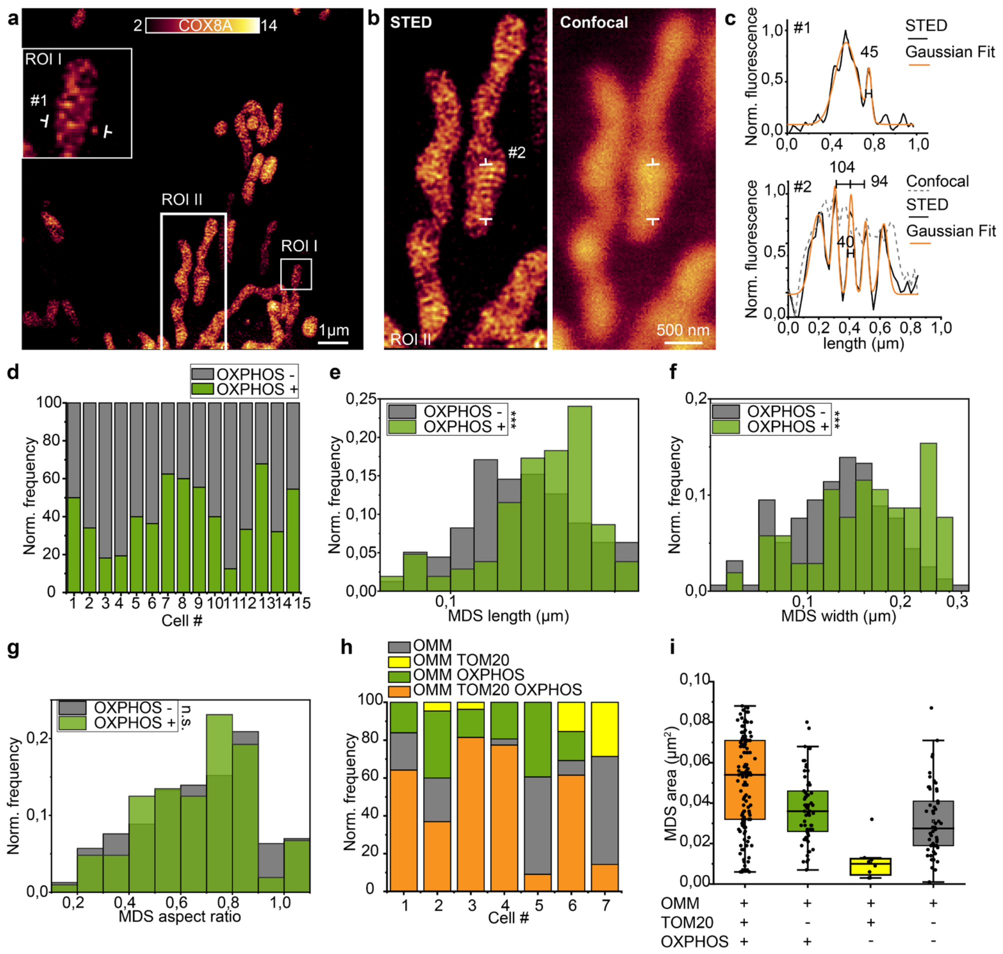

Supplementary Figure 4. MDS are characterized by different sizes and protein compositions.

(a) STED image of the mitochondrial inner membrane, labelled via the exogenous expression of COX8A full-length protein fused to the SNAP-tag and covalently bound to the SiR-647-BG ligand (COX8A). The inset (ROI I) shows a small vesicle next to the tip of a mitochondrion. See Supplementary Movie 4.

(b) Zoom-in (ROI II) of one mitochondrion where cristae invaginations are visible in STED but not in the confocal comparison.

(c) Line profiles measured across #1 and #2 in (a) and (b), showing the vesicle FWHM (45 nm) and the size and distances of cristae, respectively.

(d) Bar plot showing the ratio of MDSs with respect to the presence of OXPHOS, grouped per cell, highlighting the high cell-to-cell variability. N= 15 cells. Relative to Fig. 4d.

(e) Distribution of MDS length, (f) width and (g) aspect ratio, divided into $OXPHOS^{+}$ and $OXPHOS^{-}$ MDSs. N = 15 cells; MDSs: $OXPHOS^{-}$ = 158; $OXPHOS^{+}$ = 104. KS test: $P_{length} = 3.16\times10^{-4}$; $P_{width} = 7.94\times10^{-5}$; $P_{ar} = 0.7$. Relative to Fig. 4d.

(h) Bar plot showing the ratio of MDSs with respect to the presence of OXPHOS and TOM20, grouped per cell (Cell# 1-7), highlighting the high cell-to-cell variability relative to the box plot in Fig. 4g.

(i) Box plot showing the area of MDSs, grouped by their composition. Each data point represents one MDSs. N = 7 cells; MDSs = 257. For the box plot the center line represents the median, the box spans the interquartile range (IQR; 25th to 75th percentiles) and whiskers extend to 1.5× IQR.

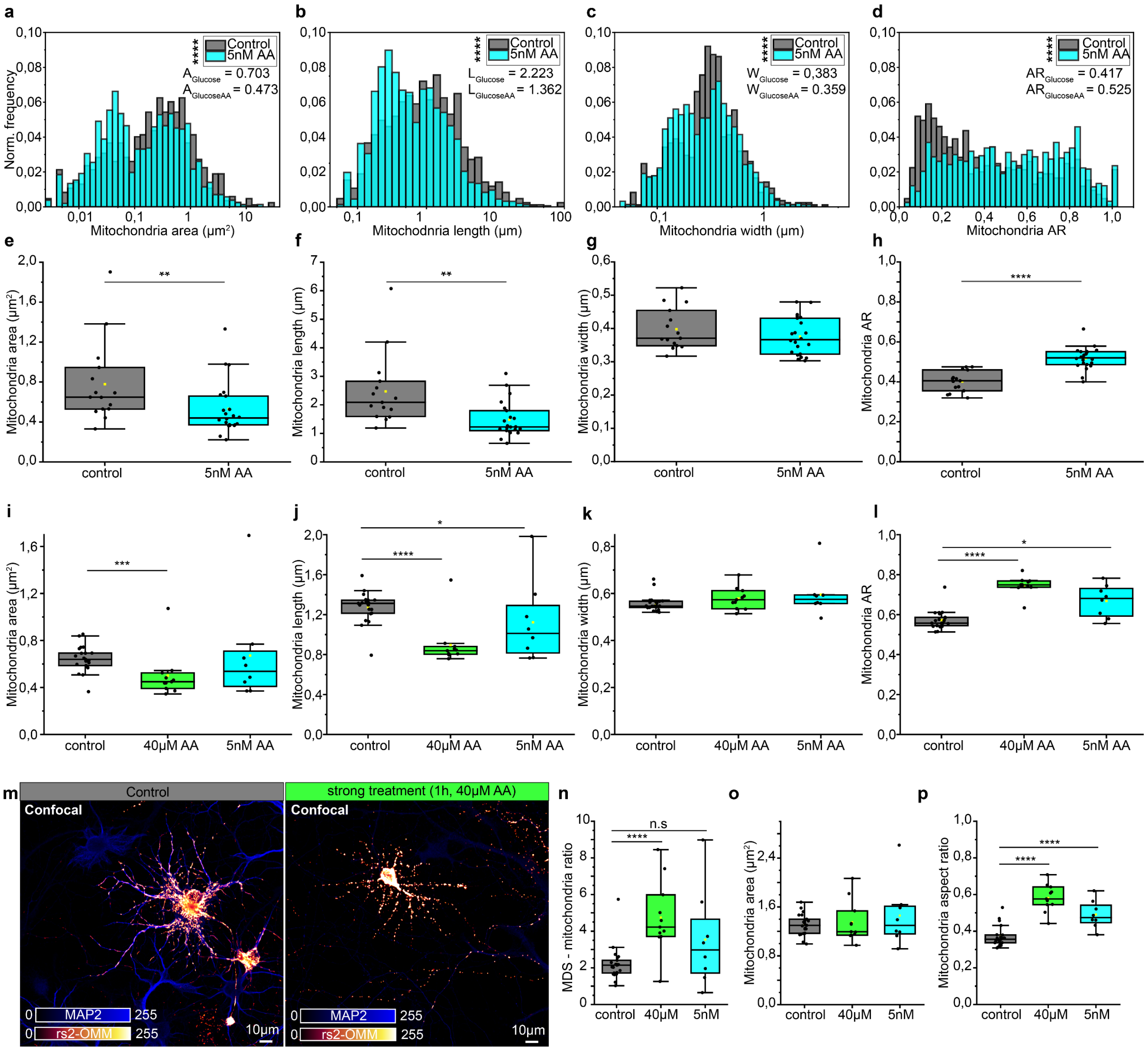

Supplementary Figure 5. Perturbation of mitochondria respiration and its effect.

(a) - (d) Histogram distribution of mitochondria area, length, width, and aspect ratio for cells grown in glucose (control, grey) and in 5nM AA for 6h (AA, cyan). Each datapoint represents one mitochondrion or MDS. KS test: $p_{area} = 8.87\times10^{-10}$; $p_{length} = 8.87\times10^{-10}$; $p_{width} = 8.87\times10^{-10}$; $p_{ar} = 8.87\times10^{-10}$; N = 2417 ($N_{control}$ = 847, N_{AA} = 1570), 3 independent experiments.

(e) - (h) Box plot of mitochondria area, length, width, and aspect ratio for cells grown in glucose (control, grey) and in 5 nM AA for 6 h (AA, cyan). Each datapoint represents one cell, 3 independent experiments. N = 37 (N_C = 15, N_{AA} = 22). KS test: $p_{area} = 0.008$; $p_{length} = 0.002$; $p_{width} = 0.54$; $p_{ar} = 2.84\times10^{-7}$.

(i) - (l) Box plot of mitochondria area, length, width, and aspect ratio for cells grown in glucose (control, grey), 40 µM for 1 h (green) and 5 nM AA for 6 h (cyan). Each data point represents one cell, 3 independent experiments.

$N = 40$ ($N_C = 21$, $N_{40\mu M} = 11$; $N_{5nM} = 8$). KS test: (I) $p_{C\text{-}40\mu M} = 1.23\times10^{-4}$; $p_{C\text{-}5nM} = 0.13$; (J) $p_{C\text{-}40\mu M} = 4.45\times10^{-6}$; $p_{C\text{-}5nM} = 0.024$; (K) $p_{C\text{-}40\mu M} = 0.066$; $p_{C\text{-}5nM} = 0.147$; (L) $p_{C\text{-}40\mu M} = 1.86\times10^{-7}$; $p_{C\text{-}5nM} = 0.024$.

(m) Confocal images of two representative cells grown in control (Glucose) and 40 µM AA for 1h. Mitochondria (Halo-OMM, red hot) and dendrites (MAP2 immunostaining, blue) were shown.

(n) - (p) Box plots of the MDSs number per mitochondria, the mitochondria area and aspect ratio for cells grown in glucose (control, grey), 40µM AA for 1h (green) and 5nM AA for 6h (cyan). Each datapoint represents one cell. $N = 40$ ($N_C = 21$, $N_{40\mu M} = 11$; $N_{5nM} = 8$) from three independent experiments. KS: (n) $p_{C\text{-}40\mu M} = 4.45\times10^{-6}$; $p_{C\text{-}5nM} = 0.12$; (o) $p_{C\text{-}40\mu M} = 0.60$; $p_{C\text{-}5nM} = 0.46$; (p) $p_{C\text{-}40\mu M} = 4.45\times10^{-6}$; $p_{C\text{-}5nM} = 0.002$.

For the box plots (e-l) and (n-p) the center line represents the median, the box spans the interquartile range (IQR; 25th to 75th percentiles) and whiskers extend to 1.5× IQR.

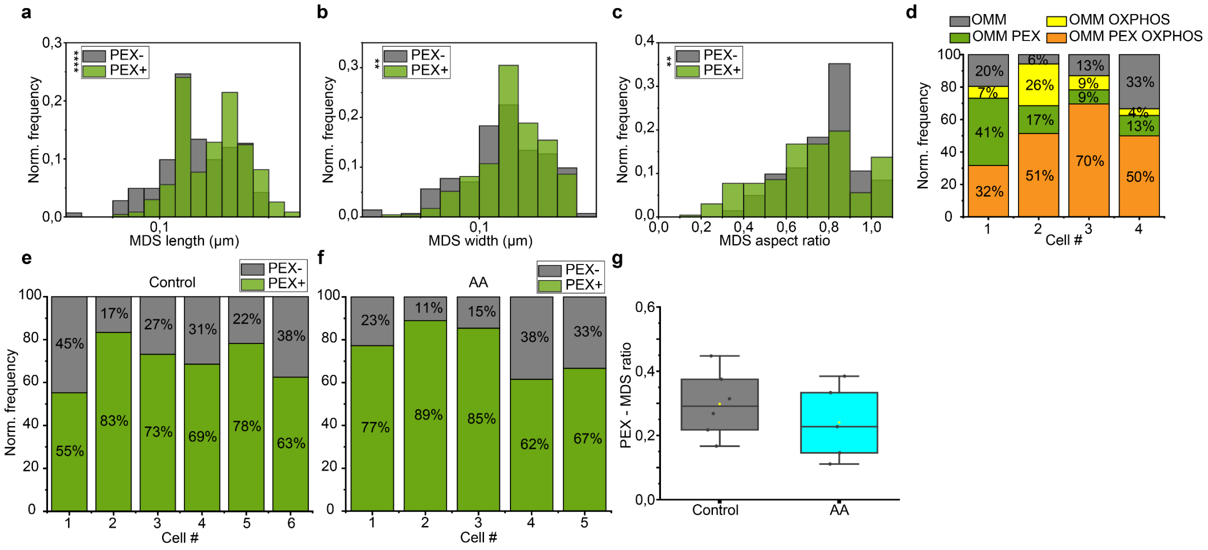

Supplementary Figure 6. Characterization of MDSs interacting with peroxisomes at basal and stress conditions.

(a) - (c) Histogram distribution of MDSs length, width, and aspect ratio, divided into PEX^+ and PEX^-. N = 6 cells. (PEX^- = 142; PEX^+ = 233). KS test: $p_{length} = 2.41\times10^{-4}$; $p_{width} = 0.005$; $p_{ar} = 0.002$.

(d) Relative abundance of MDSs types with respect to PEX14 and OXPHOS complexes, grouped per cell, highlighting the high cell-to-cell variability and reporting the statistic relative to the box plot shown in Fig. 6g.

(e) (f) Bar plot showing the ratio of MDSs with respect to PEX14, grouped per cell, for control and AA mild treatment (5nM, 6h).

(g) Box plot of the ratio between the PEX^- MDSs and the total MDSs number for control and AA treated cells, showing no differences in the number of PEX^+ MDSs upon AA treatment. Each data point represents one cell. For the box plot the center line represents the median, the box spans the interquartile range (IQR; 25th to 75th percentiles) and whiskers extend to 1.5× IQR.

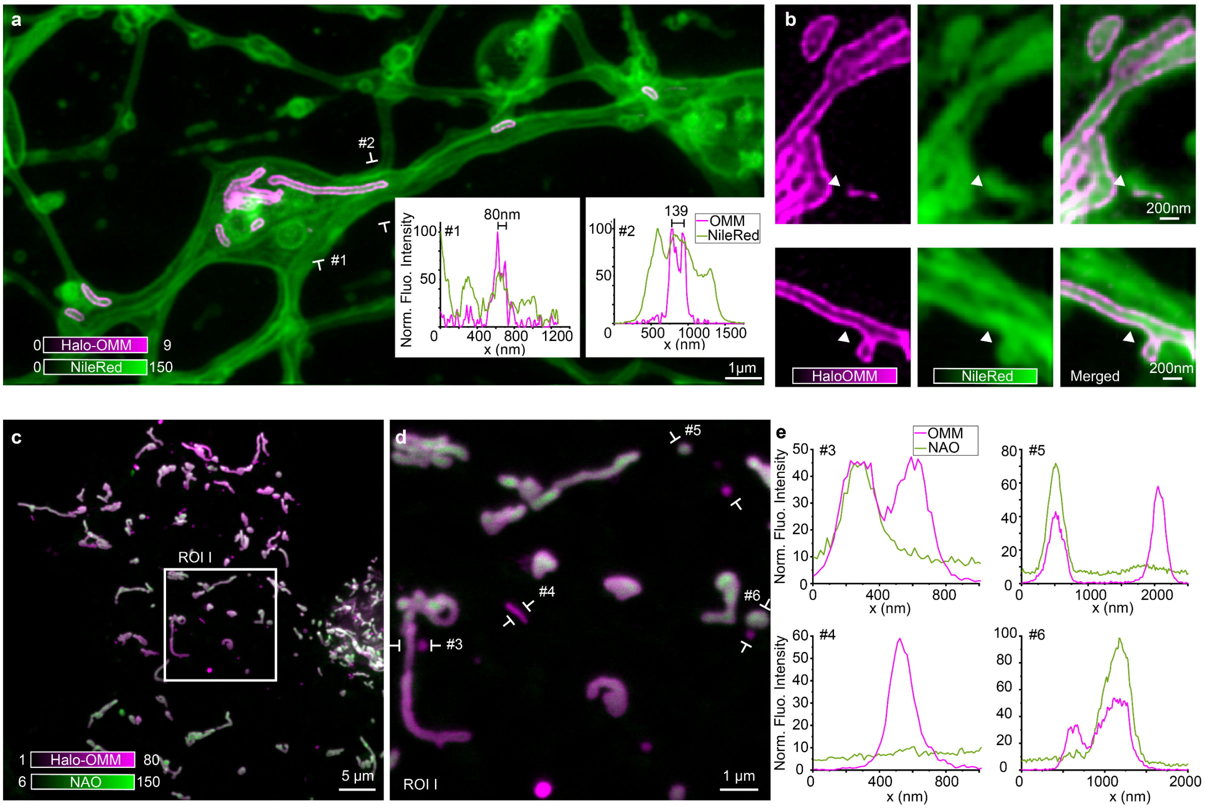

Supplementary Figure 7. MDSs colocalize with lipids.

(a) Two-colour STED image of neuronal filaments, labelled for Halo-OMM (magenta) and lipids (Nile Red dye, green). The inset shows line profiles measured across #1 and #2, showing the overlap of the two labels both at vesicles and mitochondria sites, respectively.

(b) Two-colour STED images showing examples of a stick and vesicle protrusion where OMM and lipid signals (Nile Red, green) are overlapping.

(c) Image of the neuronal cell, labelled for the Halo-OMM (magenta) in STED and for cardiolipin (NAO dye, green) in confocal.

(d) Zoom-in of the region highlighted in C (ROI I) showing examples of MDSs which do not show a cardiolipin content.

(e) Line profiles across #1, #2, #3 and #4 indicated in D.

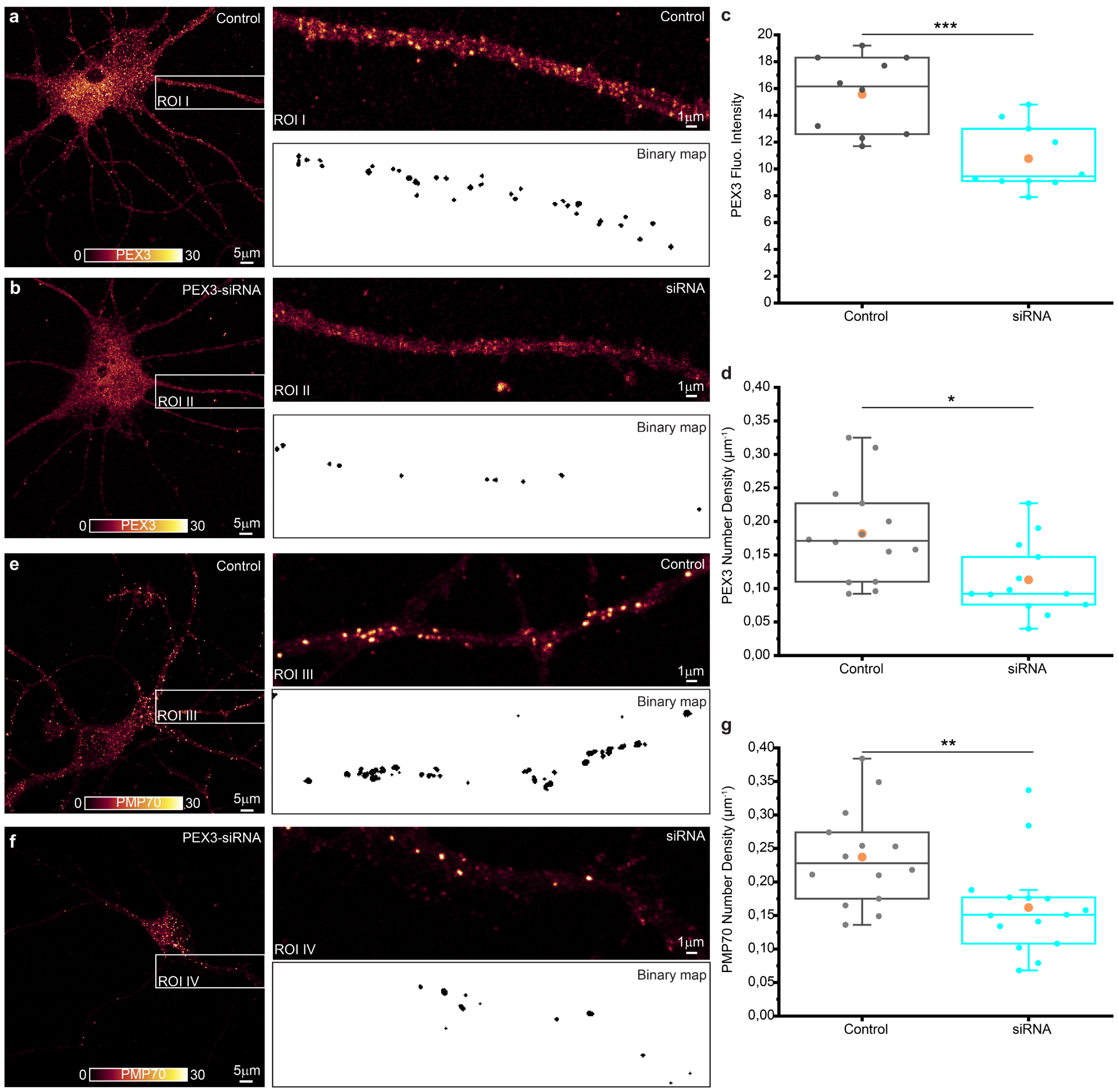

Supplementary Figure 8. PEX3 knock-down.

(a) Representative example of a neuronal cell where exogenous PEX3 proteins are fluorescently labelled via immunostaining with an anti-PEX3 specific antibody. ROI I shows a neuronal filament where PEX3 puncta are visualized in the confocal scan (Control) and subsequently masked based on their intensity (binary map).

(b) Representative example of a neuronal cell treated for 48h with 3X siRNAs molecules to downregulate the translation of PEX3 fluorescently labelled via immunostaining with an anti-PEX3 specific antibody. ROI II shows a neuronal filament where PEX3 puncta are visualized in the confocal scan (siRNA) and subsequently masked based on their intensity (Binary map).

(c) Box plot of the PEX3 fluorescence intensity measured in control (grey) and in siRNA treated cells (cyan). Each datapoint represent one cell. Two-sample Student's t-test p = $7.68462*10^{-4}$.

(d) Box plot of the PEX3 number density measured in control (grey) and in siRNA treated cells (cyan). Each datapoint represent one cell. Two-sample Student's t-test p = 0.01075.

(e) Representative example of a neuronal cell where exogenous PMP70 proteins are fluorescently labelled via immunostaining with an anti-PMP70 specific antibody. ROI I shows a neuronal filament where PMP70 puncta are visualized in the confocal scan (control) and subsequently masked based on their intensity (Binary map).

(f) Representative example of a neuronal cell treated for 48h with 3X siRNAs molecules to downregulate the translation of PEX3 fluorescently labelled via immunostaining with an anti-PMP70 specific antibody. ROI II shows a neuronal filament where PMP70 puncta are visualized in the confocal scan (siRNA) and subsequently masked based on their intensity (Binary map).

(g) Box plot of the PMP70 number density measured in control (grey) and in siRNA treated cells (cyan). Each datapoint represent one cell. Two-sample Student's t-test p = 0.00903.

For the box plots in (c, d, g) the center line represents the median, the box spans the interquartile range (IQR; 25th to 75th percentiles) and whiskers extend to 1.5× IQR.

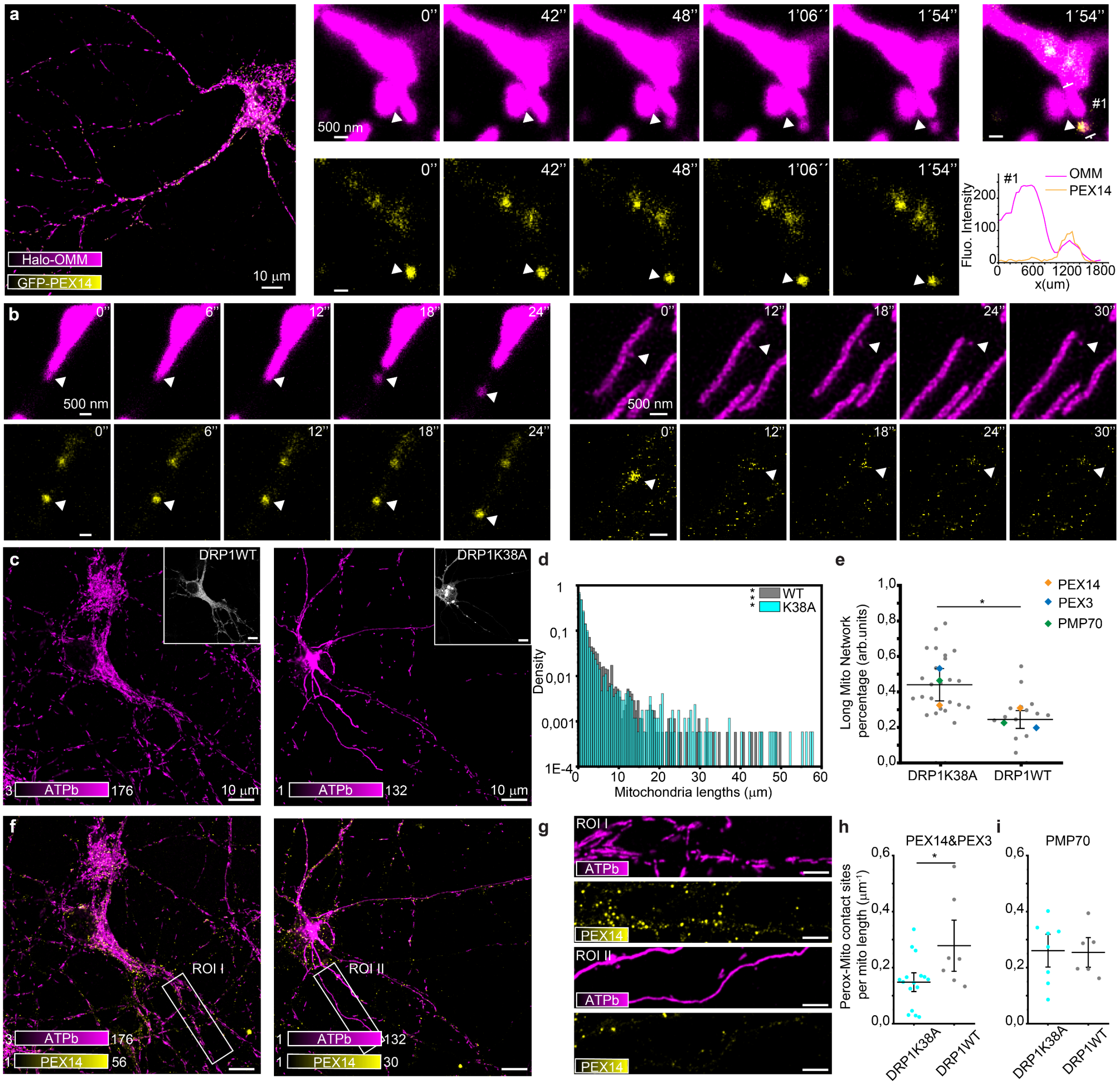

Supplementary Figure 9. Pre-peroxisomes formation and perturbation of the *de novo* peroxisome biogenesis process.

(a) Representative two-colour live confocal time-lapse of mitochondria (Halo-OMM, magenta) and peroxisomes (PEX14, yellow). Zoom-in of five frames, mitochondria top and peroxisomes bottom, shows a mitochondrial protrusion forming at the tip and detaching with PEX14. Last frame shows both colors with a line profile #1, at the detachment site. The movie has been recorded for roughly two minutes at a frame rate of 6 frames/minute.

(b) Two examples of mitochondrial protrusions colocalizing with PEX14 and MDS formation, imaged with confocal (left, ~1.5minute, 6 frames/minute) and STED (right, ~30sec, 6 frames/minute) timelapse.

(c) Two representative examples of neuronal mitochondrial network (ATPb immunostaining) exogenously expressing DRP1WT (left, inset: DRP1WT-mCherry) or DRP1K38A (right, inset: DRP1K38A-mCherry), highlighting the mitochondria elongation upon DRP1K38A expression.

(d) Histogram distribution of the mitochondrial lengths measured in neurons expressing DRP1WT (grey) or DRP1K38A (cyan). Data from 3 independent experiments. $N_{DRP1WT} > 4000$, $N_{DRP1K38A} > 400$. KS test: $p = 1.79 \times 10^{-5}$.

(e) Quantification of the sum of lengths of mitochondria longer than 10 μm divided by the length of all mitochondria. Grey dots are data averaged per cell: $N_{DRP1WT} = 15$, $N_{DRP1K38A} = 26$, from 3 independent experiments. The horizontal lines represent the means, the error bars represent the standard error of the means, and the colored diamonds represent the means per experiment: Exp1-PEX14, yellow; Exp2-PEX3, blue; Exp3-PMP70, green.

(f) Same cells shown in (c), showing the mitochondrial network (ATPb immunostaining, magenta) and peroxisomes (PEX14, yellow).

(g) Representative ROIs from (f) showing the decrease amount of peroxisomal structures detected in connection with mitochondria upon expression of DRP1K38A with respect to the control cells (DRP1WT).

(h) Quantification of the overlapping sites per mitochondrial length between peroxisomal puncta labeled with the pre-peroxisomal biogenesis markers PEX14 and PEX3 and mitochondrial longer than 10 μm measured in DRP1K38A or DRP1WT expressing cells. Each datapoint is one cell, same data as in (e).

(i) Quantification of the number of overlapping sites per mitochondrial length between peroxisomal puncta labeled with PMP70 and mitochondrial longer than 10 μm measured in DRP1K38A or DRP1WT expressing cells. Each datapoint is one cell, same data as in (e).

Supplementary Notes

Supplementary Note 1. STED imaging does not affect mitochondrial membrane potential or ROS production.

To rule out potential side effects of STED light doses on mitochondrial membrane potential, neurons were monitored under either STED (continuous or interval) or confocal illumination (Supplementary Figure 3a). In all three imaging modalities, both STED and confocal recordings showed that about 10% of mitochondria lost TMRE labelling after 25 imaging frames (Supplementary Figure 3b). We did not observe any alterations in TMRE intensity or mitochondrial area during STED recordings (Supplementary Figure 3 c–d). Control experiments demonstrated that STED illumination had a very low impact on mitochondrial membrane potential and morphology during our imaging experiments.

We checked for side effects of MitoSOX on mitochondrial bioenergetics by labelling neurons with either MitoSOX alone (Supplementary Figure 3e–f) or in combination with MitoTracker Green (mtGreen), a dye that causes cell phototoxicity upon continuous high-intensity blue-light illumination[1] (Supplementary Figure 3g–h). We measured the fluorescence intensity of the two dyes at different time points (1–10 frames) in a confocal scan. The mean intensity of mitochondria labelled with MitoSOX alone remained constant over 10 frames (Supplementary Figure 3f). However, when mtGreen was present, the fluorescence intensity of MitoSOX increased over time, while that of mtGreen decreased, indicating a correlation between mtGreen photobleaching and increased mitochondrial ROS (Supplementary Figure 3h)[2, 3]. We therefore conclude that low doses of MitoSOX alone do not affect mitochondrial ROS production in neuronal cells.

Supplementary Note 2. Perturbation of PEX3 and DRP1 affects peroxisome *de novo* biogenesis

To investigate the role of MDSs in *de novo* peroxisome biogenesis, we perturbed key proteins involved in peroxisome biogenesis and mitochondrial dynamics (Supplementary Figure 8 and 9).

First, we perturbed peroxisomal biogenesis 3 (PEX3), a core component of the peroxisome biogenesis machinery required for the assembly of membrane vesicles prior to the import of matrix proteins (Supplementary Figure 8). PEX3 was shown to be involved in the process of pre-peroxisome formation via MDVs. We performed transient knockdown of PEX3 in neuronal cells using RNA silencing. Three commercially available siRNAs targeting three different regions of the PEX3 transcript (s222855; s222856; s222857 ThermoFisher Scientific) were transfected into DIV6 neurons. Following the manufacturer's protocol, siRNAs were transfected into DIV6 neuronal cultures,

and PEX3 downregulation was assessed 24- and 48-hours post-transfection by immunostaining. Silencing of PEX3 resulted in a marked reduction of PEX3 signal intensity compared to untreated controls. Quantitative analysis of PEX3 puncta intensity at 48 hours post-transfection revealed a significant decrease in endogenous PEX3 levels (two-sample *t*-test, $P = 7.68 \times 10^{-4}$, equal variance) relative to non-transfected neurons (Supplementary Figure 8a–c), confirming efficient suppression of PEX3 synthesis. A significant reduction of PEX3-puncta density was measured upon silencing (two-sample *t*-test, $P = 0.01075$) (Supplementary Figure 8d). A comparable decrease was observed in the density of mature peroxisomes, labeled with the peroxisomal membrane protein PMP70 (two-sample *t*-test, $P = 0.00903$) (Supplementary Figure 8e–g). Together, these findings demonstrate that transient PEX3 knockdown effectively impairs *de novo* peroxisome formation in neuronal cells, as reflected by the reduced abundance of both PEX3 and mature peroxisomal structures.

Then, to investigate the role of DRP1 we expressed the dominant-negative DRP1K38A mutant, which inhibits GTP binding and hydrolysis, thereby impairing mitochondrial constriction and division (Supplementary Figure 9). DRP1K38A expression induced the elongation of mitochondrial tubules and the overall network. Analysis of the spatial relationship between mitochondria and pre-peroxisomal markers (PEX3 and PEX14) revealed a significant reduction in the number of pre-peroxisomal puncta contacting mitochondria, whereas interactions with mature peroxisomes (labelled with PMP70) were unchanged. These results indicate that disruption of DRP1 selectively impairs the association of pre-peroxisomal structures with mitochondria, highlighting its role in regulating *de novo* peroxisome biogenesis mediated by MDSs.

Supplementary Information References

1. Magidson, V. & Khodjakov, A. Circumventing photodamage in live-cell microscopy. *Methods Cell Biol* **114**, 545-560 (2013).
2. Icha, J., Weber, M., Waters, J.C. & Norden, C. Phototoxicity in live fluorescence microscopy, and how to avoid it. *Bioessays* **39** (2017).
3. Tosheva, K.L., Yuan, Y., Matos Pereira, P., Culley, S. & Henriques, R. Between life and death: strategies to reduce phototoxicity in super-resolution microscopy. *J Phys D Appl Phys* **53**, 163001 (2020).
