# Supplementary material for: Quantitative optical nanoscopy of mitochondrial-derived vesicles in neurons classifies pre-peroxisomal and clearing organelles
Source: Nat Commun. 2026 Jan 8;17:419. doi: 10.1038/s41467-025-68160-y (PMC12796351; doi:10.1038/s41467-025-68160-y)
Supplement: Supplementary file 2 — Descriptions of Additional Supplementary Files [file 41467_2025_68160_MOESM2_ESM.pdf]

Descriptions of Additional Supplementary Files

File Name: Supplementary Movie 1:
Description:
Mitochondria dynamics inside a neuronal process. STED timelapse imaging of mitochondria showing a fission event and the formation of a mitochondrial derived vesicle between the newly formed mitochondria. 18 timepoints with an interval of 60 seconds. Scale bar, 500 nm. This movie refers to the data presented in Supplementary Fig. 1j.

File Name: Supplementary Movie 2:
Description:
Lateral protrusion and MDSs formation. STED time-lapse imaging of the lateral protrusion of the mitochondrial membrane followed by the formation of a mitochondrial-derived vesicle. 11 time-points with an interval of 30 seconds. Scale bar, 500 nm. This movie refers to the data presented in Supplementary Fig. 1k.

File Name: Supplementary Movie 3:
Description:
Mitochondria-ER interactions. Two-color STED time-lapse imaging of fast dynamic interactions between mitochondria (magenta) and ER tubules (cyan). ER tubules are forming a ring-like structure around a mitochondria nanotunnel, and they are also present at site of vesicle formation. The movie is recorded for 70 time points with an interval of 1.3 seconds. Scale bar, 1 mm. This movie refers to the data presented in Supplementary Fig. 1m -n.

File Name: Supplementary Movie 4:
Description:
COX8A and OMP25 dynamics. Two -color STED time -lapse imaging of mitochondria outer membrane (magenta) and COX8A (green) recorded for 12 time points with an interval of 30 seconds, showing inner and outer mitochondrial membrane dynamics. There are different examples of mitochondria protrusions and vesicles with either both or outer membrane only. Scale bar, 500 nm. This movie refers to the data presented in Supplementary Fig. 4a.

File Name: Supplementary Movie 5:
Description:
ER marks the site of MDS lateral release. Two -color STED time - lapse imaging of mitochondria (magenta) and ER (cyan) recorded for 7 time points with an interval of 30 seconds, showing ER tubules delimiting the site of a mitochondrial vesicle release. Scale bar, 500 nm. This movie refers to the data presented in Fig. 9a.

File Name: Supplementary Movie 6:
Description:
ER structures are surrounding MDS tip release. Two -color STED time -lapse imaging of mitochondria (magenta) and ER (cyan) recorded for 10 time points with an interval of 30 seconds, showing ER structures surrounding a mitochondrial tubule extension which ends with the release of an MDV at the tubule tip, upon its retraction. Scale bar, 500 nm. This movie refers to the data presented in Fig. 9b.
