# Supplementary material for: Quantitative optical nanoscopy of mitochondrial-derived vesicles in neurons classifies pre-peroxisomal and clearing organelles
Source: Nat Commun. 2026 Jan 8;17:419. doi: 10.1038/s41467-025-68160-y (PMC12796351; doi:10.1038/s41467-025-68160-y)
Supplement: Supplementary file 9 — Reporting Summary [file 41467_2025_68160_MOESM9_ESM.pdf]

nature portfolio

Corresponding author(s): Ilaria Testa
Last updated by author(s): Oct 27, 2025

Reporting Summary

Nature Portfolio wishes to improve the reproducibility of the work that we publish. This form provides structure for consistency and transparency in reporting. For further information on Nature Portfolio policies, see our Editorial Policies and the Editorial Policy Checklist.

Statistics

For all statistical analyses, confirm that the following items are present in the figure legend, table legend, main text, or Methods section.

n/a	Confirmed	
☐	☒	The exact sample size (*n*) for each experimental group/condition, given as a discrete number and unit of measurement
☐	☒	A statement on whether measurements were taken from distinct samples or whether the same sample was measured repeatedly
☐	☒	The statistical test(s) used AND whether they are one- or two-sided<br>*Only common tests should be described solely by name; describe more complex techniques in the Methods section.*
☒	☐	A description of all covariates tested
☒	☐	A description of any assumptions or corrections, such as tests of normality and adjustment for multiple comparisons
☐	☒	A full description of the statistical parameters including central tendency (e.g. means) or other basic estimates (e.g. regression coefficient) AND variation (e.g. standard deviation) or associated estimates of uncertainty (e.g. confidence intervals)
☐	☒	For null hypothesis testing, the test statistic (e.g. *F*, *t*, *r*) with confidence intervals, effect sizes, degrees of freedom and *P* value noted<br>*Give P values as exact values whenever suitable.*
☒	☐	For Bayesian analysis, information on the choice of priors and Markov chain Monte Carlo settings
☒	☐	For hierarchical and complex designs, identification of the appropriate level for tests and full reporting of outcomes
☒	☐	Estimates of effect sizes (e.g. Cohen's *d*, Pearson's *r*), indicating how they were calculated

Our web collection on statistics for biologists contains articles on many of the points above.

Software and code

Policy information about availability of computer code

Data collection	STED and confocal data were acquired either with open-source hardware and acquisition control software ImSwitch in Python, developed in the lab and available at https://github.com/ImSwitch/ImSwitch, or Imspector, or Leica Imaging Software.
Data analysis	Data analysis was performed in Fiji/ImageJ, Python 3, Matlab, OriginLab 2020, Ilastik software, as specified in the Methods section. Custom code and scripts for the various parts of the analysis in ImageJ and MATLAB, together with example data for running the scripts, are available at https://github.com/jonatanalvelid/mitography-public and as a versioned release in Zenodo at https://doi.org/10.5281/zenodo.17831561.

For manuscripts utilizing custom algorithms or software that are central to the research but not yet described in published literature, software must be made available to editors and reviewers. We strongly encourage code deposition in a community repository (e.g. GitHub). See the Nature Portfolio guidelines for submitting code & software for further information.

Data

Policy information about availability of data

All manuscripts must include a data availability statement. This statement should provide the following information, where applicable:

- Accession codes, unique identifiers, or web links for publicly available datasets
- A description of any restrictions on data availability
- For clinical datasets or third party data, please ensure that the statement adheres to our policy

No restriction on data availability. All code and raw data are available upon request.

Research involving human participants, their data, or biological material

Policy information about studies with human participants or human data. See also policy information about sex, gender (identity/presentation), and sexual orientation and race, ethnicity and racism.

Reporting on sex and gender	n/a
Reporting on race, ethnicity, or other socially relevant groupings	n/a
Population characteristics	n/a
Recruitment	n/a
Ethics oversight	n/a

Note that full information on the approval of the study protocol must also be provided in the manuscript.

Field-specific reporting

Please select the one below that is the best fit for your research. If you are not sure, read the appropriate sections before making your selection.

☒ Life sciences ☐ Behavioural & social sciences ☐ Ecological, evolutionary & environmental sciences

For a reference copy of the document with all sections, see nature.com/documents/nr-reporting-summary-flat.pdf

Life sciences study design

All studies must disclose on these points even when the disclosure is negative.

Sample size	No statistical test was applied beforehand to assess the sample size. The number of datapoints was determined for each experiment so to provide enough statistical power considering sample variability and maximized when applicable
Data exclusions	Experiments in primary neuronal cultures were excluded when, from morphological evaluation, the cell healthiness was assessed as compromised.
Replication	Each experiments was independently replicated multiple times in several sample replicas (as reported in the corresponding figure legend) and the biological findings were reproduced.
Randomization	Samples, such as neurons derived from the same litter isolation and cell lines plated from the same propagation batch, were randomly allocated into groups prior to the experiment.
Blinding	Blinding was not possible during data collection, however the semi-automated analysis enabled concurrent data processing

Reporting for specific materials, systems and methods

We require information from authors about some types of materials, experimental systems and methods used in many studies. Here, indicate whether each material, system or method listed is relevant to your study. If you are not sure if a list item applies to your research, read the appropriate section before selecting a response.

Materials & experimental systems

n/a	Involved in the study	
☐	☒	Antibodies
☒	☐	Eukaryotic cell lines
☒	☐	Palaeontology and archaeology
☐	☒	Animals and other organisms
☒	☐	Clinical data
☒	☐	Dual use research of concern
☒	☐	Plants

Methods

n/a	Involved in the study	
☒	☐	ChIP-seq
☒	☐	Flow cytometry
☒	☐	MRI-based neuroimaging

Antibodies

Antibodies used	anti-PEX14 (Biosite, 10594-1-AP-20, 1:200); Total OXPHOS Rodent WB Antibody Cocktail (Abcam, ab110413, 1:200); anti-TOM20

Antibodies used	(Santa Cruz Biotechnology, sc-11415, 1:50 dilution); anti-MAP2 (Abcam, ab5392, 1:2000); anti-TFAM (Abcam, ab13160, 1:200); FluoTag®-X4 anti-GFP (NanoTag, N0304-Ab580 or N0304-Ab635P-L); anti-PMP70 (Abcam, ab85550, 1:200); anti-mCherry (Abcam, ab167453, 1:200); anti-Drp1 (Cell Signaling Tech, D6C7 #8570); anti-PEX3 (Atlas Antibodies, HPA042830); anti-rabbit Alexa594 (Thermo Fisher Scientific, A-11037, 1:200 dilution); anti-mouse Alexa594 (Thermo Fisher Scientific, A-21203, 1:200 dilution); anti-rabbit STAR RED (Abberior, 2-0012-011-9, 1:200 dilution); anti-mouse STAR RED (Abberior, 2-0002-011-2, 1:200 dilution); anti-mouse AlexaFluor488 (Thermo Fisher Scientific, A-11001, 1:200 dilution), anti-Chicken AlexaFluor488 (Abcam, ab150173, 1:200 dilution).
Validation	When applicable, antibodies were tested in primary neurons or cell lines verifying colocalization with an other marker or with a previously validated antibody against the same protein. Most of the primary antibodies employed were previously used in several publications. DOI: 10.1038/s41598-018-24119-2; doi: 10.1038/srep04983; DOI: 10.1073/pnas.1301820110; DOI: 10.1126/science.aao6047.

Animals and other research organisms

Policy information about studies involving animals; ARRIVE guidelines recommended for reporting animal research, and Sex and Gender in Research

Laboratory animals	Sprague Dawley rat embryos, embryonic day 18 (RjHan:SD , JanvierLabs).
Wild animals	n/a
Reporting on sex	Embryos were collected randomly from the pregnant rats without prior determination of sex.
Field-collected samples	n/a
Ethics oversight	ll experiments were performed in accordance with animal welfare guidelines set forth by Karolinska Institutet and were approved under the Ethical Permit nr 2645-2021 by the Swedish Board of Agriculture (Jordbruksverket).

Note that full information on the approval of the study protocol must also be provided in the manuscript.

Plants

Seed stocks	n/a
Novel plant genotypes	n/a
Authentication	n/a
